# Investigating reliable amyloid accumulation in Centiloids: Results from the AMYPAD Prognostic and Natural History Study

**DOI:** 10.1002/alz.13761

**Published:** 2024-04-04

**Authors:** Ariane Bollack, Lyduine E. Collij, David Vállez García, Mahnaz Shekari, Daniele Altomare, Pierre Payoux, Bruno Dubois, Oriol Grau‐Rivera, Mercè Boada, Marta Marquié, Agneta Nordberg, Zuzana Walker, Philip Scheltens, Michael Schöll, Robin Wolz, Jonathan M. Schott, Rossella Gismondi, Andrew Stephens, Christopher Buckley, Giovanni B. Frisoni, Bernard Hanseeuw, Pieter Jelle Visser, Rik Vandenberghe, Alexander Drzezga, Maqsood Yaqub, Ronald Boellaard, Juan Domingo Gispert, Pawel Markiewicz, David M. Cash, Gill Farrar, Frederik Barkhof, AMYPAD consortium

**Affiliations:** ^1^ Centre for Medical Image Computing (CMIC) Department of Medical Physics and Bioengineering University College London London London UK; ^2^ Department of Radiology and Nuclear Medicine Amsterdam UMC Amsterdam The Netherlands; ^3^ Clinical Memory Research Unit Department of Clinical Sciences Lund University Malmö Sweden; ^4^ Amsterdam Neuroscience, Brain Imaging VU University Amsterdam Amsterdam The Netherlands; ^5^ Barcelonaβeta Brain Research Center (BBRC), Pasqual Maragall Foundation Barcelona Spain; ^6^ Universitat Pompeu Fabra Barcelona Spain; ^7^ Instituto de investigaciones médicas Hospital del Mar (IMIM) Barcelona Spain; ^8^ Neurology Unit Department of Clinical and Experimental Sciences University of Brescia Brescia Italy; ^9^ Department of Nuclear Medicine Imaging Pole Toulouse University Hospital Toulouse France; ^10^ Toulouse NeuroImaging Center Université de Toulouse Inserm UPS CHU Purpan Pavillon Baudot Place du Docteur Joseph Baylac Toulouse France; ^11^ Department of Neurology Salpêtrière Hospital AP‐HP Sorbonne University Paris France; ^12^ Ace Alzheimer Center Barcelona – Universitat Internacional de Catalunya Barcelona Spain; ^13^ CIBERNED Network Center for Biomedical Research in Neurodegenerative Diseases National Institute of Health Carlos III Madrid Spain; ^14^ Department of Neurobiology Care Sciences and Society, Center for Alzheimer Research, Division of Clinical Geriatrics, Karolinska Institutet Stockholm Sweden; ^15^ Theme Inflammation and Aging, Karolinska University Hospital, Karolinska Institutet Stockholm Sweden; ^16^ Division of Psychiatry University College London London UK; ^17^ Essex Partnership University NHS Foundation Trust, The Lodge Wickford UK; ^18^ Alzheimer Center and Department of Neurology Amsterdam Neuroscience, VU University Medical Center, Alzheimercentrum Amsterdam Amsterdam The Netherlands; ^19^ Wallenberg Centre for Molecular and Translational Medicine, The University of Gothenburg Gothenburg Sweden; ^20^ Department of Psychiatry and Neurochemistry Institute of Neuroscience and Physiology, The Sahlgrenska Academy, University of Gothenburg, Sahlgrenska University Hospital Gothenburg Sweden; ^21^ Department of Neurodegenerative Disease UCL Institute of Neurology London UK; ^22^ IXICO Plc London UK; ^23^ Dementia Research Centre, UCL Queen Square Institute of Neurology London UK; ^24^ Life Molecular Imaging, GmbH Berlin Germany; ^25^ GE HealthCare Buckinghamshire UK; ^26^ Department of Neurology Institute of Neuroscience, Université Catholique de Louvain, Cliniques Universitaires Saint‐Luc Brussels Belgium; ^27^ Gordon Center for Medical Imaging Department of Radiology Massachusetts General Hospital Boston Massachusetts USA; ^28^ WELBIO Department WEL Research Institute Wavre Belgium; ^29^ Alzheimer Center Limburg, School for Mental Health and Neuroscience, Maastricht University Maastricht The Netherlands; ^30^ Laboratory for Cognitive Neurology, LBI – KU Leuven Brain Institute Leuven Belgium; ^31^ Department of Nuclear Medicine University Hospital Cologne, Universitätsklinikums Köln Köln Germany; ^32^ Molecular Organization of the Brain, Institute for Neuroscience and Medicine, INM‐2), Forschungszentrum Jülich GmbH Jülich Germany; ^33^ German Center for Neurodegenerative Diseases (DZNE) Bonn Germany; ^34^ Nuclear Medicine and Molecular Imaging, University Medical Center Groningen, University of Groningen Groningen The Netherlands; ^35^ Centro de Investigación Biomédica en Red de Bioingeniería, Biomateriales y Nanomedicina, Instituto de Salud Carlos III Madrid Spain; ^36^ Computer Science and Informatics, School of Engineering, London South Bank University London UK; ^37^ Queen Square Institute of Neurology, University College London London UK; ^38^ UK Dementia Research Institute at University College London London UK

**Keywords:** Alzheimer's, amyloid, Centiloid, longitudinal PET, quantification, reliable accumulation

## Abstract

**INTRODUCTION:**

To support clinical trial designs focused on early interventions, our study determined reliable early amyloid‐β (Aβ) accumulation based on Centiloids (CL) in pre‐dementia populations.

**METHODS:**

A total of 1032 participants from the Amyloid Imaging to Prevent Alzheimer's Disease–Prognostic and Natural History Study (AMYPAD‐PNHS) and Insight46 who underwent [^18^F]flutemetamol, [^18^F]florbetaben or [^18^F]florbetapir amyloid‐PET were included. A normative strategy was used to define reliable accumulation by estimating the 95^th^ percentile of longitudinal measurements in sub‐populations (*N*_PNHS_ = 101/750, *N*_Insight46_ = 35/382) expected to remain stable over time. The baseline CL threshold that optimally predicts future accumulation was investigated using precision‐recall analyses. Accumulation rates were examined using linear mixed‐effect models.

**RESULTS:**

Reliable accumulation in the PNHS was estimated to occur at >3.0 CL/year. Baseline CL of 16 [12,19] best predicted future Aβ‐accumulators. Rates of amyloid accumulation were tracer‐independent, lower for APOE ε4 non‐carriers, and for subjects with higher levels of education.

**DISCUSSION:**

Our results support a 12–20 CL window for inclusion into early secondary prevention studies. Reliable accumulation definition warrants further investigations.

## 1 BACKGROUND

Accumulation of cerebral amyloid beta (Aβ) plaques is a key early marker of Alzheimer's disease (AD) pathophysiology, starting decades before the first symptoms appear.^1^ Sharp reductions of Aβ were observed in recent lecanemab and donanemab trials in patients with early cognitive impairment who were selected to be amyloid positive.^2^, ^3^, ^4^ However, it is unknown how these anti‐Aβ therapies will affect individuals before symptom onset. This long preclinical phase is the focus of recent secondary prevention trials with anti‐Aβ therapy such as the A4 and AHEAD 3‐45 studies,^5^, ^6^ which aim to remove incipient aggregates or limit future accumulation.^7^, ^8^ Longitudinal positron emission tomography (PET) studies enable the detection and quantification of small changes in Aβ over time, which is an important outcomes of these trials.^9^ This is supported by recent studies that showed that the rate of Aβ accumulation, rather than baseline burden, improved prediction of cognitive decline in preclinical populations.^10^, ^11^, ^12^ Identifying early subjects that will accumulate Aβ in the near future can help select those most likely to reach amyloid positivity and benefit from treatment now that successful therapies are becoming available.^13^

While rates of change in Aβ deposition are commonly measured using annualized rates of change in standard uptake value ratio (SUVr), the Centiloid scale (CL) is increasingly being used to minimize differences arising from multiple centers and tracers, including in the latest phase III trials of aducanumab,^14^ lecanemab^2^ and donanemab.^3^, ^4^, ^15^ The CL approach was introduced in 2015 as a means of calibrating measures of Aβ deposits to a tracer‐independent unbounded scale, where 0 (value characteristic of young healthy controls) and 100 (typical AD subjects) act as anchor points.^16^ Longitudinal trajectories of Aβ accumulation have been previously described,^1^, ^17^, ^18^ with more recent studies using the CL scale to characterize the pathophysiological rates of Aβ increase.^19^, ^20^, ^21^, ^22^, ^23^, ^24^, ^25^ Although visual reading is currently the approved method for image interpretation in clinical practice (requiring a binary classification of normal/abnormal), the importance of quantifying PET measurement and its uncertainty has been highlighted in the latest Radiological Society of North America Quantitative Imaging Biomarkers Alliance (QIBA) profile.^26^ Quantitative information generated by CE‐marked software can also now be used as an adjunct to visual interpretation.^27^, ^28^, ^29^ Hence, there is a need for estimates of longitudinal PET changes in CL units that account for measurement uncertainty and intrinsic variability to determine reliable Aβ accumulation trajectories.

Quantification of amyloid burden in “absolute” units can be leveraged into the definition of threshold differentiating stages of amyloid pathology that are comparable across centers and tracers. So far, using mostly cross‐sectional data, various CL thresholds and windows have been established based on histopathology,^30^, ^31^, ^32^ visual read,^19^, ^33^, ^34^, ^35^, ^36^, ^37^ agreement with other amyloid biomarkers,^38^ and disease stage.^30^, ^39^ These thresholds have been established to reflect the earliest signs of the presence of Aβ compared to post‐mortem studies (∼10–12 CL), and compared to visual reads (∼16–26 CL) (summary in Pemberton et al.^9^). Finally using longitudinal data from cognitively unimpaired individuals at baseline, the optimal baseline threshold for predicting an abnormal increase in Aβ using PiB was found to be 17.5 CL in the Harvard Aging Brain Study (HABS), 15.0 CL in the Australian Imaging, Biomarker & Lifestyle Flagship Study of Ageing (AIBL),^25^ and 19 ± 7 CL in the Mayo Clinic Study of Aging (MCSA).^24^ A [^18^F]florbetapir (FBP) threshold of 16.7 CL was also defined using data from the Alzheimer's Disease Neuroimaging Initiative (ADNI).^25^ However, the definition and robustness of such a threshold for [^18^F]flutemetamol (FMM) and [^18^F]florbetaben (FBB) remains to be explored.

To further support clinical trial designs that are focused on early intervention, the present study aimed to characterize early Aβ accumulation based on CL units for FMM and FBB in a pre‐dementia population, by (1) estimating the variability of longitudinal CL measurements in a population expected to remain stable over time in order to define reliable accumulation beyond measurement error, (2) establishing the baseline CL threshold that optimally predicts future accumulation, and (3) describing the rates of Aβ accumulation across the whole population and investigating their relation to visual read status over time.

## 2 METHODS

### 2.1 Cohorts

#### 2.1.1 AMYPAD‐PNHS

The present work uses clinical and imaging data from the European Amyloid Imaging to Prevent Alzheimer's Disease (AMYPAD, https://amypad.eu/) Prognostic and Natural History Study (PNHS),^40^ downloaded from the Alzheimer's disease data initiative workbench (version v202306, https://doi.org/10.5281/zenodo.8017084). The AMYPAD consortium was initiated in 2016 as part of the Innovative Medicine Initiative‐Alzheimer's disease platform.^41^ The AMYPAD‐PNHS is a prospective, multi‐center, pan‐European study, focused on using amyloid PET to further our understanding of AD in its pre‐dementia phase. Clinical and imaging data were collected from 11 parent cohorts with similar characteristics (EudraCT 2018‐002277‐22). Inclusion criteria were as follows: the participant (1) should be non‐demented (i.e., Clinical Dementia Rating global score (CDR) ≤ 0.5), (2) older than 50 years of age, (3) able to undergo MRI and PET‐acquisition, and (4) active or previously enrolled in a Sponsor‐approved parent cohort.

The current analysis included participants who underwent longitudinal PET imaging (*N *= 750 with one PET follow‐up, time interval: 3.4 ± 1.9 years; *N *= 96 with two PET follow‐ups, time interval at follow‐up 2: 5.2 ± 0.7 years).

#### 2.1.2 Insight46

In order to validate estimates of reliable accumulation in a separate cohort, 282 subjects from Insight46, a prospective neuroscience sub‐study of the MRC National Survey of Health and Development,^42^ with baseline and follow‐up dynamic PET‐MR scans (follow‐up time: 2.4 ± 0.2 years) acquired with [^18^F]florbetapir were included. All study members were born in the same week of 1946 and the majority were cognitively normal.

**RESEARCH IN CONTEXT**

1. **Systematic review**: The authors reviewed the literature using traditional sources, focusing on dementia‐related research studies involving longitudinal amyloid positron emission tomography (PET), specifically the ones using the Centiloid (CL) scale. While many studies report the change in amyloid deposition over time, the rate of change that can reliably be considered an increase in amyloid remains unclear.
2. **Interpretation**: Our findings suggest that a rate of change of 3 CL/year or more can be considered reliable accumulation. They also support a CL window of 12–20 for inclusion into early secondary prevention studies.
3. **Future directions**: Our results should be further validated in datasets representing the whole AD *continuum*. The notion of reliable accumulation should be investigated according to the tracer, and should consider potential changes in scanner or tracer.

### 2.2 PET acquisition

#### 2.2.1 AMYPAD PNHS

PET data were acquired using either FMM (*N *= 481, 64%) or FBB (*N *= 269, 36%). In accordance with the tracers’ image acquisition guidance, four frames of 5 minutes were acquired starting at 90 minutes post‐injection of 185 MBq ± 10% of FMM^28^ or 300 MBq ± 10% of FBB.^27^ An image harmonization protocol was implemented to ensure that quantitative metrics were comparable across centers,^43^ resulting in a final effective image resolution of 8 mm across scanners. No partial volume correction was applied.

#### 2.2.2 Insight46

Images were acquired on the same Biograph mMR 3T PET/MRI scanner (Siemens Healthcare, Erlangen). The full study protocol is described elsewhere.^42^ In short, 370 MBq of FBP was injected intravenously, after which PET data were acquired continuously for ∼60 min. Only static analysis was used in this study and relied on the last ∼10 minutes of scanning (from 50 to 60 minutes). Attenuation correction was performed using a pseudo‐CT generated from the MR.^44^ Images were smoothed with a 4 mm Gaussian kernel and no partial volume correction was applied.

### 2.3 Image processing

#### 2.3.1 AMYPAD PNHS

First, the quality of the scans was manually assessed. Scans that were deemed to be of sufficient quality were then processed using IXICO's in‐house fully automated MR‐based PET workflow. Briefly, PET frames were co‐registered to create an average image that was aligned to the subject's T1 weighted (T1w) image. Global cortical average and whole cerebellum uptakes were computed from the corresponding GAAIN masks (http://www.gaain.org/centiloid‐project) from which SUVr values were derived. Following the reference pipeline,^16^ SUVr values were converted into appropriate tracer‐specific CL metrics.^45^

#### 2.3.2 Insight46

The Centiloid pipeline used in Insight46 also followed the reference pipeline by Klunk et al.^16^ Details of the implementation can be found in Coath et al.^46^

### 2.4 Visual reads

PNHS images were classified as either positive (VR+: binding in one or more cortical brain regions unilaterally, as well as striatum for FMM) or negative (VR−: predominantly white matter uptake) by certified nuclear physicians or radiologists according to criteria defined by the manufacturers (Life Molecular Imaging for NeuraCeq and GE HealthCare for Vizamyl). Based on VR status over time, subjects were categorized as Stable VR− (VR− at baseline and follow‐up), Converters (VR− at baseline and VR+ at the first or the second follow‐up), or Stable VR+ (VR+ at baseline and follow‐up). Twelve participants had a VR+ at baseline and VR− during follow‐ups.

### 2.5 Statistical analyses

R version 4.3.0 (R Program for Statistical Computing) was used for all statistical analyses.

#### 2.5.1 Definition of reliable accumulation

To assess longitudinal CL uncertainty, a subset of the overall study population was identified to form a reference group, with individuals expected not to accumulate Aβ over time.

For the PNHS, inclusion criteria for the reference group were as follows: baseline CL negative (<12), baseline and follow‐up VR negative, CSF Aβ42/40, or Aβ42 negative (measures and thresholds were either cohort‐specific or based on assay specifications), and CSF p‐tau negative, resulting in the selection of 101 individuals. All CSF measures were taken within 1 year of the baseline PET acquisition or during follow‐ups.

This approach was replicated in Insight46 by subjects according to the following criteria: at follow‐up, CSF Aβ42/40 value in the top quartile or normality and normal CSF ptau181 (≤57 pg/mL using cut‐off from the manufacturer and further validated^47^), no mild cognitive impairment or major brain disorder at baseline (based clinical consensus criteria^48^), yielding 16 individuals.

The definition of reliable accumulation and therefore the classification of individuals as Aβ‐accumulators or non‐accumulators was based on an individual annualized rate of change (ARC) being greater than the 95^th^ percentile of the mean ARC in the reference population. Alternatively, we also investigated the use of Gaussian mixture modeling (GMM, *k *= 2 Gaussian distributions) to define reliable accumulation and Aβ‐accumulators as individuals with an ARC greater than the 99^th^ percentile of the first component (corresponding to the mode with the lowest ARC).

#### 2.5.2 Precision‐recall analysis

The baseline CL threshold that best predicts future Aβ accumulation and VR conversion was established through precision‐recall analysis, maximizing the F1‐score (i.e., the harmonic mean of the precision and recall). In order to inform secondary prevention trials, a similar analysis excluding individuals with a positive VR at baseline was performed. Bootstrap resampling was used both to optimize the threshold (500 repetitions) and derive its 95% confidence interval (CI; validation using out‐of‐sample predictions from 1000 repetitions). Three additional scenarios were investigated by setting a minimum precision and recall of 0.7 and a minimum specificity of 0.9. A precision‐recall analysis was preferred to a receiver operating characteristic analysis as it is better suited for data with imbalanced classes.^49^

#### 2.5.3 Characterizing longitudinal trajectories

Longitudinal trajectories of Aβ accumulation were modeled by fitting a linear mixed effect model (LME) to the whole cohort (*lmer* 1.1.33 package in R) with CL as the outcome measure. The first model included the effect of time, group (i.e., PNHS reference/study group) and the interaction between the two, allowing for group‐specific random effects (with an unstructured covariance matrix). For the study group, fitting included random intercepts and slopes, while for the reference group we allowed random intercepts only in order not to overfit the model.

The effect of known risk factors and tracer on both baseline and changes in CL over time were then investigated in the following order: baseline age, APOE‐ε4 carriership, PET tracer, sex, and education (as categorical variable: compulsory/upper‐secondary/post‐secondary education). These factors and their interaction with time were kept in subsequent models if they improved the fit of the model, assessed using the corrected Akaike information criterion, and if their effect on CL burden was statistically significant.

We then investigated whether CL load at baseline and the annualized CL accumulation differed across VR status over time (i.e., Stable VR−/Converters/Stable VR+) and cognitive state (i.e., Cog: cognitively unimpaired (CDR = 0)/cognitive impaired (CDR ≥ 0.5)). For this analysis, only Converters from VR− to VR+ were included.

Bootstrap resampling from 1000 samples was used to derive 95% confidence intervals of model estimates.

### 2.6 Data and code availability

Data used in the preparation of this article were obtained from the AMYPAD PNHS dataset, (version v202306, https://doi.org/10.5281/zenodo.8017084). The R code use for the analysis can be found on Zenodo (https://doi.org/10.5281/zenodo.10808658).

## 3 RESULTS

### 3.1 Demographics

Demographic characteristics for the whole cohort are summarized in Table 1. The PNHS dataset comprised 750 pre‐dementia subjects with longitudinal amyloid‐PET imaging. Participants had a median age of 65 years (range = 49 to 96 years), 57% were females, 41% were APOE‐ε4 carriers, and 18% had baseline VR+ scan. Individuals with FMM acquisitions had a lower baseline age compared to those with FBB acquisitions (65.3 ± 8.0 vs. 66.4 ± 6.9 years, *p *< 0.001), as well as a higher proportion of APOE‐ε4 carriers (FMM = 46%, FBB = 33%, *χ* ^2^ = 24.52, *p *< 0.001).

**TABLE 1:** Demographic characteristics of the PNHS data, split by tracer.

Variable	Overall, *N* = 750^a^	Florbetaben, *N* = 269^a^	Flutemetamol, *N *= 481^a^	*p*‐value
Age, years	65.7 (7.6)	66.4 (6.9)	65.3 (8.0)	<0.001^b^
Gender				0.2^c^
Male	324/750 (43%)	107/269 (40%)	217/481 (45%)	
Female	426/750 (57%)	162/269 (60%)	264/481 (55%)	
APOE ε4 alleles (% carriers)				0.002^c^
0	429/735 (58%)	173/259 (67%)	256/476 (54%)	
1	270/735 (37%)	73/259 (28%)	197/476 (41%)	
2	36/735 (5%)	13/259 (5%)	23/476 (5%)	
(Missing)	15	10	5	
VR category				0.12^d^
Stable VR−	562/750 (75%)	211/269 (78%)	351/481 (73%)	
Converters+ (VR− > VR+)	56/750 (7%)	17/269 (6%)	39/481 (8%)	
Converters− (VR+ > VR−)	12/750 (2%)	1/269 (0%)	11/481 (2%)	
Stable VR+	120/750 (16%)	40/269 (15%)	80/481 (17%)	
Education, years				0.003^c^
Compulsory	133/750 (18%)	54/269 (20%)	79/481 (16%)	
Upper‐secondary	218/750 (29%)	58/269 (22%)	160/481 (33%)	
Post‐secondary	399/750 (53%)	157/269 (58%)	242/481 (50%)	
CDR				0.007^c^
0 – Normal	701/747 (94%)	242/267 (91%)	459/480 (96%)	
0.5 – Very mild	46/747 (6%)	25/267 (9%)	21/480 (4%)	
(Missing)	3	2	1	
MMSE	29.2 (1.0)	29.2 (1.0)	29.2 (1.0)	0.6^b^
(Missing)	59	20	39	
*N* timepoints				<0.001^c^
2	654/750 (87%)	173/269 (64%)	481/481 (100%)	
3	96/750 (13%)	96/269 (36%)	0/481 (0%)	
Time interval follow‐up 1, years	3.00 (2.10, 4.00)	2.20 (2.00, 2.90)	3.40 (2.60, 4.20)	
Time interval follow‐up 2, years	5.30 (5.20, 5.40)	5.30 (5.20, 5.40)	–	
Baseline CL	14.1 (24.6)	12.6 (25.3)	15.1 (24.1)	0.002^b^
ARC, CL/years	1.5 (3.3)	1.8 (3.7)	1.4 (3.0)	0.4^b^

Abbreviations: APOE, apolipoprotein E; ARC, annualized rate of change, computed as (CL_max(follow‐up)_ – CL_baseline_)/dt.; CL, Centiloid; CDR, Clinical Dementia Rating Global Score, MMSE, Mini‐Mental State Examination; VR, visual read.

^a^ Mean (SD); *n*/*N* (%).

^b^ Wilcoxon rank sum test.

^c^ Pearson's chi‐squared test.

^d^ Fisher's exact test.

The whole cohort was then split into a reference subset with individuals unlikely to accumulate amyloid over the duration of the study (*N *= 101; including *N*_FMM _= 86 and *N*_FBB _= 15; mean follow‐up time 3.2 years, SD = 0.9, range = 1.4 to 5.2 years) and an exploratory set (*N *= 649; including *N*_FMM _= 395 and *N*_FBB _= 254).

In the reference group, the mean baseline amyloid load was 2.4 ± 5.7 CL, compared to 14.1 ± 23.3 CL in the exploratory group, with no significant difference between tracers (*p *= 0.2). No difference in age, sex, APOE‐ε4 carriership, or years of education was observed across tracers in this reference group.

### 3.2 Defining reliable Aβ accumulation

**FIGURE 1:**
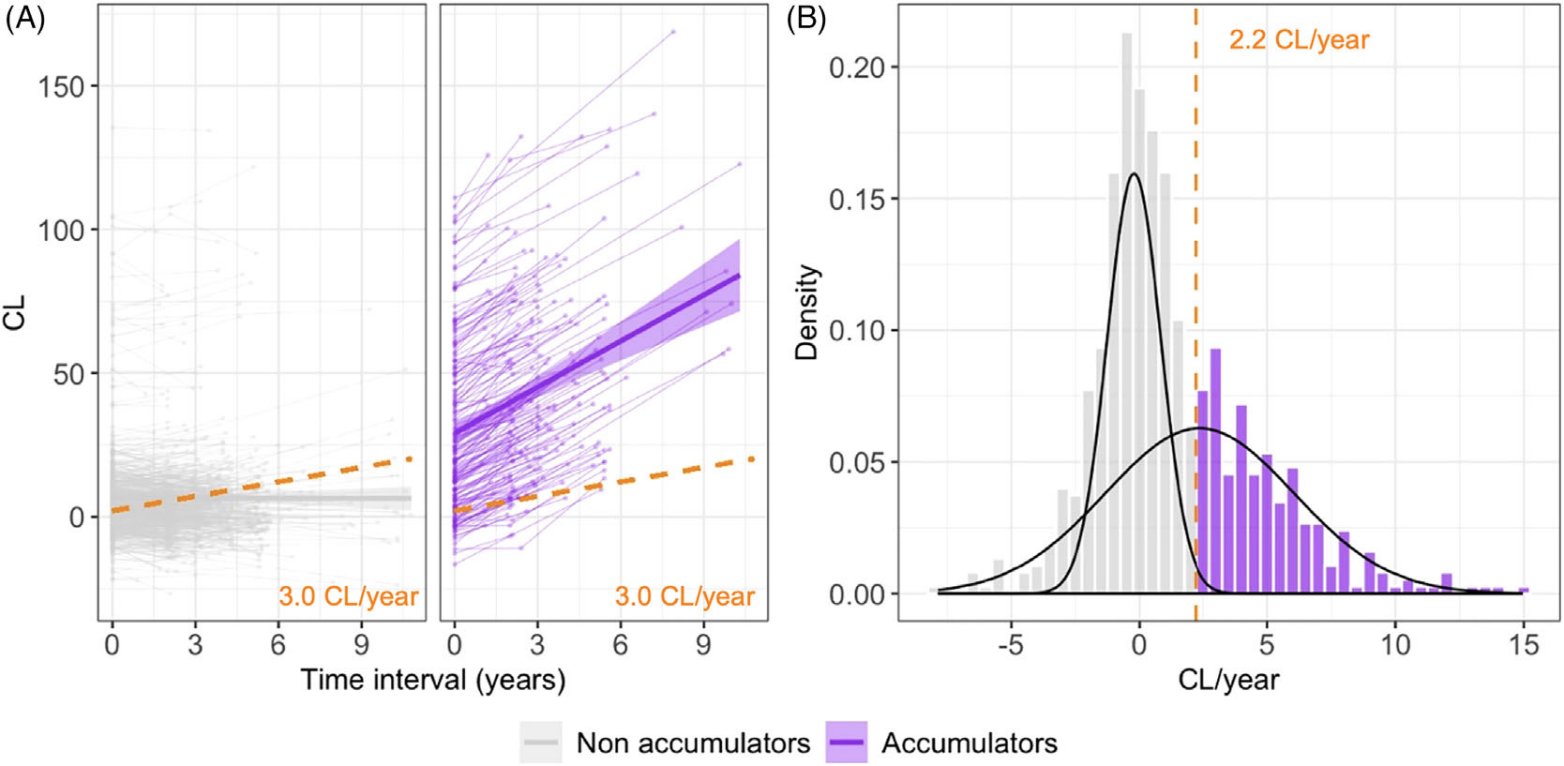
Definition of reliable accumulation using two approaches. (A) Reliable accumulation based on the 95^th^ percentile of the annualized CL rate of change in a reference group (i.e., >3.0 CL/year), represented by the orange dotted lines. The plot displays longitudinal CL trajectories within the PNHS exploratory subset, for Aβ‐accumulators (individuals that showed reliable accumulation, in purple) and non‐accumulators (in gray). (B) Reliable accumulation based on gaussian mixture modeling (*k* = 2) using the whole PNHS cohort. The orange vertical line represents the 99th percentile of the first Gaussian distribution and corresponds to 2.2 CL/year. ARC, annualized rates of change; CL, Centiloid; PNHS, Prognostic and Natural History Study.

**FIGURE 2:**
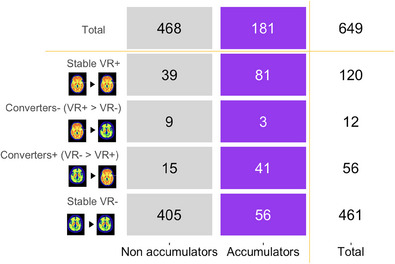
Number of subjects in each category within the exploratory cohort. Aβ‐Accumulators based on the 95^th^ percentile of the annualized CL rate of change in a reference group (i.e., >3.0 CL/year). Aβ, amyloid‐β; CL, Centiloid; VR, visual reads.

Reliable Aβ accumulation in the PNHS was defined as an ARC greater than 3.0 CL/year, corresponding to the upper bound of the 95% CI of the ARC in the reference group (Figure 1A). Within the exploratory cohort, individuals were then categorized as Aβ‐accumulators and non‐accumulators based on whether they surpassed the threshold of reliable accumulation. According to this definition, 27.9% of individuals in the exploratory cohort were Aβ‐accumulators. As expected, this number was significantly higher in the Converters and Stable VR+ groups (44/68 = 64.7% and 81/120 = 67.5%, respectively, see count per category in Figure 2). In the Stable VR− group, still 12.1% (56/461) were classified as Aβ‐accumulators despite having a shorter follow‐up time compared to non‐accumulators (*p *= 0.006). Aβ‐accumulators were on average 3 years older than non‐accumulators (67.8 ± 7.8 vs. 64.8 ± 7.7 years, *p *= 0.001) and tended to have a higher baseline CL load (8.3 ± 14.1 CL vs. 3.0 ± 8.7 CL, *p *= 0.016). No difference in sex or APOE‐ε4 carriership was observed.

Using GMM as an alternative method to define reliable accumulation, the reliable Aβ accumulation threshold corresponded to an ARC greater than 2.2 CL/year (Figure 1B).

In Insight46, the reliable accumulation estimate was 3.7 CL/year using the 95^th^ percentile of the ARC in the reference group and 3.2 CL/year using the GMM approach. The Aβ‐accumulators had a higher percentage of APOE‐ε4 carriers (Aβ‐accumulators = 50%, non‐accumulators = 21%, *χ* ^2^ = 39.01, *p *< 0.001) and females (Aβ‐accumulators = 45%, non‐accumulators = 56%, *χ* ^2^ = 4.64, *p *= 0.04).

### 3.3 Optimal threshold to predict future Aβ‐accumulators

**FIGURE 3:**
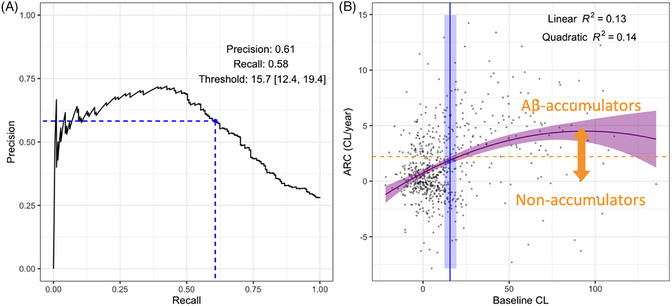
(A) Precision‐Recall curve using baseline CL load as predictor to identify Aβ‐Accumulators. In blue, the maximum F1 score corresponds to a baseline amyloid load of 15.7 [12.4, 19.4] CL; Bootstrap resampling was used both to optimize the threshold (500 repetitions) and derive its 95% confidence interval (CI; validation using out‐of‐sample predictions from 1000 repetitions). (B) ARC versus baseline CL load. The blue line represents a baseline threshold of 15.7 CL. The shaded blue area defines the boundaries of the 95% CI around the threshold. The orange line represents the limit above which subjects are considered Aβ‐Accumulators (ARC > 3.0 CL/year). The purple curve represents the data fitted with a quadratic polynomial. Aβ, amyloid‐β; ARC, annualized rate of change; CI, confidence interval; CL, Centiloid; VR, visual read.

To determine the optimal threshold to predict future Aβ accumulation, a Precision‐Recall analysis was used to classify individuals as Aβ‐accumulators or non‐accumulators (ARC > 3.0 CL/year) based on their baseline CL load. The resulting threshold and 95% CI were 15.7 [12.4, 19.4] (Figure 3). Importantly, in individuals with a baseline VR−, the threshold is lower (12.9 [8.8, 16.6] CL). FMM threshold was 17.4 [13.7, 21.4], higher than FBB 13.0 [8.5, 18.6], albeit overlapping CI. Using the GMM‐based definition of Aβ‐accumulators yields a similar but slightly lower threshold of 12.8 [9.1, 16.5] CL.

**FIGURE 4:**
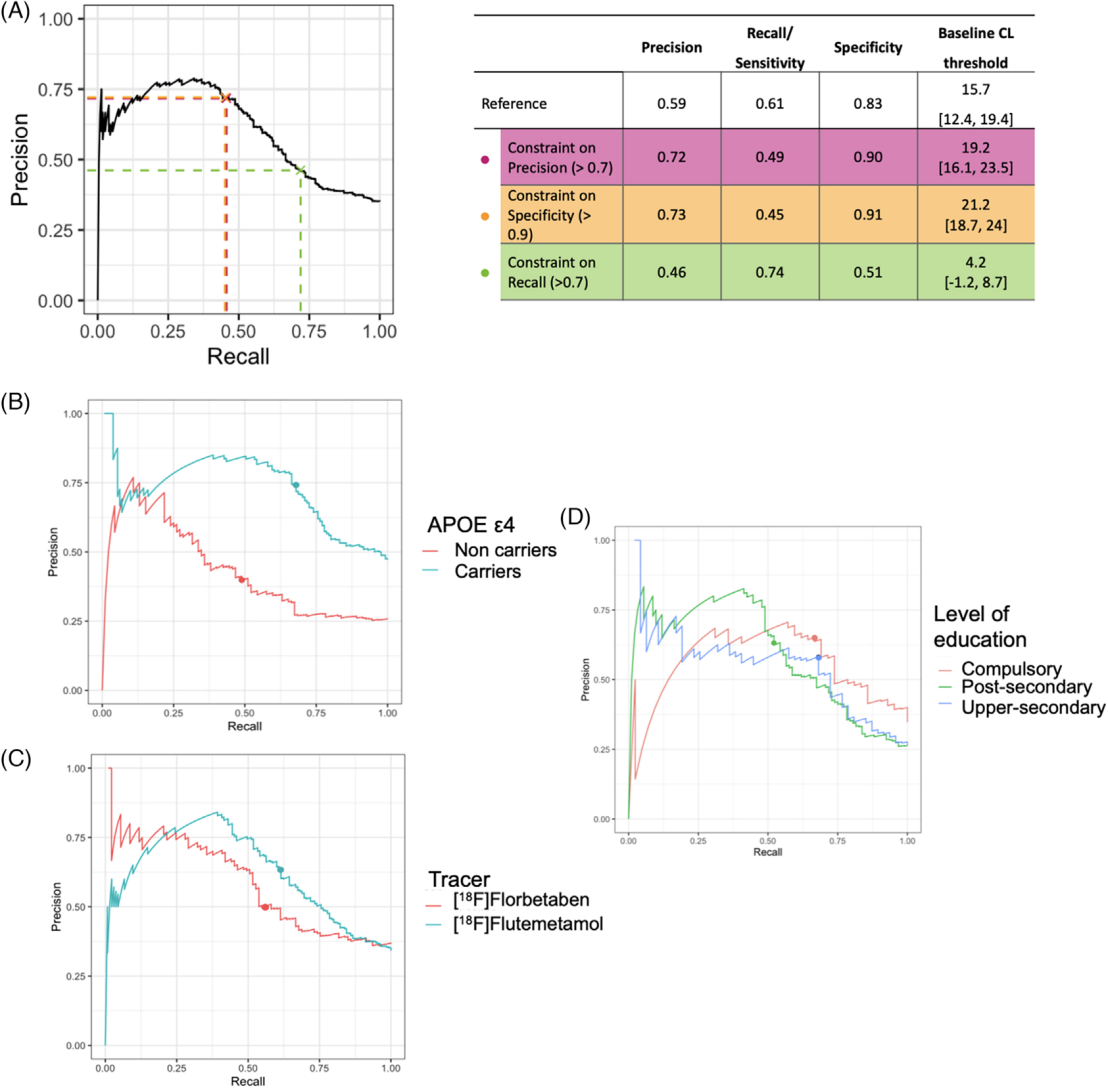
Summary of Precision‐Recall Analysis using baseline CL to predict reliable accumulation. The optimal baseline CL threshold is determined by maximizing the F1‐score. (A) Three additional scenarios were investigated by adding a constraint on precision, recall or specificity (minimum value = 0.7 for precision and recall, 0.9 for specificity). Bootstrap resampling was used both to optimize the threshold (500 repetitions) and derive its 95% confidence interval (CI; validation using out‐of‐sample predictions from 1000 repetitions). (B, C, D) Precision‐Recall curves according to APOE ε4 carriership, tracer, and level of education respectively. APOE, apolipoprotein E; AUC, area under the curve; CL, Centiloid.

Three additional scenarios were investigated by setting a minimum precision, recall and specificity of 0.7 (Figure 4A). While adding a constraint on precision and specificity produces comparable results, increasing recall at the expense of other metrics greatly lowered the threshold to 4.2 [−1.2, 8.7] CL.

Furthermore, the predictive value of baseline CL is higher in APOE‐ε4 carriers individuals (precision = 0.72; recall = 0.70, threshold: 12.4 [6.4, 15.9]) (Figure 4B) and for participants scans with FMM (precision = 0.63; recall = 0.63, threshold: 14.3 [10.5, 17.8]) compared to FBB (precision = 0.53) (Figure 4C). Finally, the sensitivity decreased and the specificity increased with higher levels of education (Figure 4D), which also resulted in higher baseline CL thresholds to predict future accumulation (threshold _compulsory_: 14.0 [6.1, 21.1]; threshold _upper‐secondary_: 15.5 [12.4, 19.6] threshold _post‐ secondary_: 18.8 [12.8, 23.7]).

### 3.4 Longitudinal Aβ‐PET trajectories

Longitudinal trajectories of amyloid accumulation were characterized using LME. The first model highlighted the differences between exploratory and reference groups, with a higher baseline CL in the former (baseline CL_exploratory_ = 14.0 [12.1, 15.8], baseline CL_reference_ = 2.3 [1.0, 3.5], *t* = −10.1, *p* < 0.001), and by definition a higher average ARC (ARC_exploratory _= 1.5 [1.3, 1.8] CL/year, ARC_reference_ = −0.2 [−0.5, 0.2], *t* = −8.4, *p* < 0.001).

In a second step, we tested the predictive value of baseline age, APOE‐ε4 carriership, PET tracer, sex, education (in this order), and their interaction with time as covariates.

**FIGURE 5:**
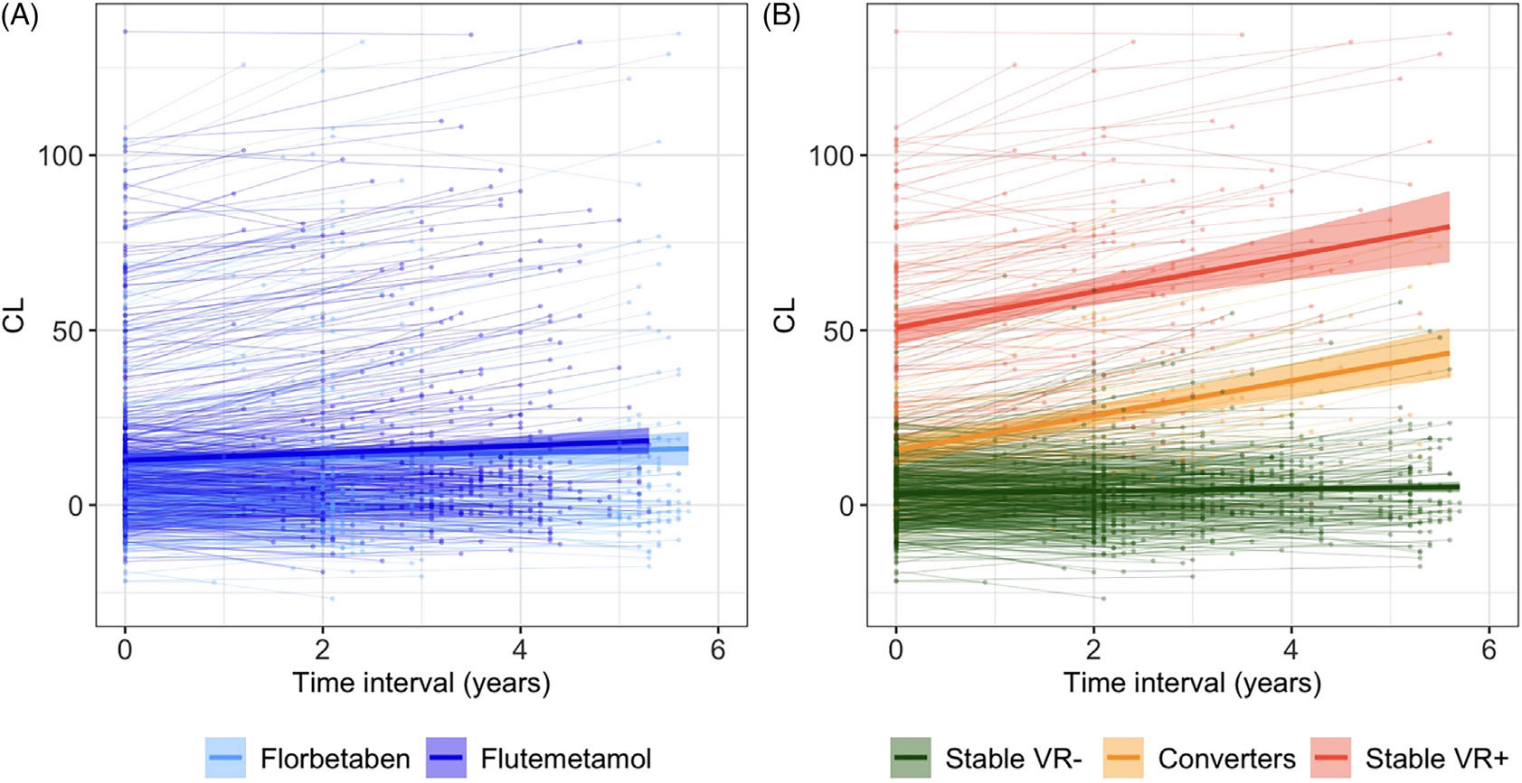
Longitudinal trajectories of amyloid accumulation (A) by tracer and (B) based on VR over time. CL, Centiloid scale; VR, visual reads.

First, baseline age and APOE‐ε4 carriership had a significant impact on baseline CL (baseline age: *p* < 0.001; APOE‐ε4 carriership: *t_ _* = 4.15, *p *< 0.001); however, only the interaction of APOE‐ε4 carriership with time was also predictive of CL accumulation over time (APOE‐ε4 carriership*time: *t_ _* = 4.21, *p* = < 0.001; baseline age*time: *t_ _* = 1.70, *p *= 0.09). Regarding the tracer, FMM baseline CL estimates were on average approximately five CL higher than the ones for FBB (*t_ _* = 3.98, *p* < 0.001). The interaction between tracer and time was not significant (*t_ _* = −1.67, *p *= 0.09) (Figure 5).

We also found no evidence of sex differences on CL load and CL over time. At this stage, the model included the following risks factors as predictors: baseline age, and APOE‐ε4 carriership and its interaction with time. Adding the level of education, however, was predictive of baseline CL load, with an amyloid burden on average 3.6 CL lower for post‐secondary education compared to compulsory level of education (post‐secondary vs. compulsory *t *= −2.31, *p *= 0.02). Our results also suggest that higher levels of education (upper‐ or post‐secondary) were indicative of slower ARC, on average −0.55 CL/year, compared to compulsory level of education (upper‐secondary vs. compulsory *t *= −2.02, *p *= 0.04; post‐secondary vs. compulsory *t *= −2.15, *p *= 0.032).

Based on these results, we included baseline age, PET tracer, APOE‐ε4 carriership and its interaction with time and the level of education as covariates for subsequent analyses (the interaction between education and time was removed from the model).

Finally, longitudinal CL trajectories across cognitive groups (i.e., cognitively unimpaired/cognitively impaired) and VR over time (i.e., Stable VR−/Converters/Stable VR+) were explored. The cognitive status of individuals based on the CDR was not predictive of CL burden. Baseline CL values and ARC were higher in Stable VR+ and Converters compared to Stable VR− (focusing on differences between Stable VR− and Converters, baseline CL: *t *= 5.34, *p *< 0.001; ARC: *t *= 14.62, *p *< 0.001), but no significant difference in ARC was found between Converters and Stable VR+ (*t *= −1.58, *p *= 0.12) (Figure 5).

## 4 DISCUSSION

**FIGURE 6:**
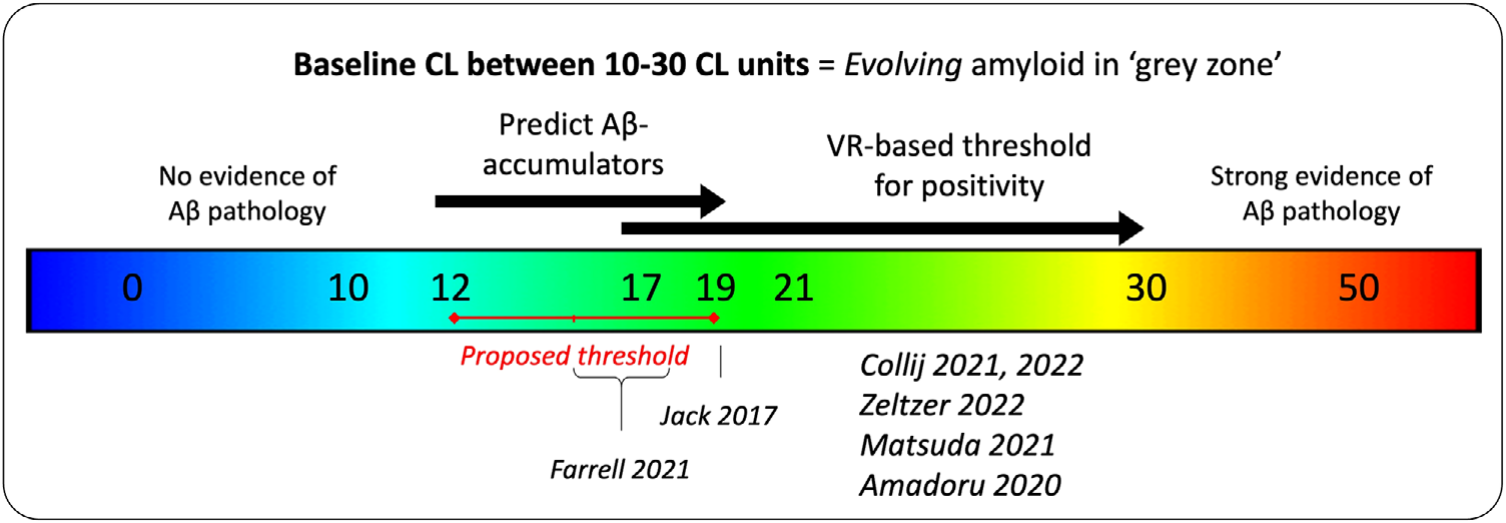
Overview of CL thresholds with a focus in the “gray zone,” between 10 and 30 CL. CL, Centiloid; VR, visual reads.

The present study characterized Aβ accumulation, as expressed in CL units based on FMM and FBB amyloid‐PET in the AMYPAD PNHS pre‐dementia population. We first estimated the variability of longitudinal CL measurements in a reference sub‐population expected to remain stable over time and defined reliable accumulation as an ARC greater than 3.0 CL/year. In a separate dataset from the Insight46 study, this was estimated at 3.7 CL/year. This notion should be further evaluated using several independent cohorts. We then established that a baseline CL threshold of 16 [12,19] could help identify future Aβ‐accumulators (Figure 6). Furthermore, in the PNHS, APOE‐ε4 carriers, and those with a lower educational background exhibited faster rates of Aβ accumulation. Notably, among participants with an initial negative VR, those who later had a VR positive scan displayed a higher amyloid burden at baseline (∼11 CL) and an increased ARC (∼4.4 CL/year) in contrast to participants who consistently tested VR negative throughout their follow‐up period.

Several strategies have been previously developed to distinguish Aβ‐accumulators from non‐accumulators, based on the SUVr and using the inflexion in between peaks of bimodal distribution of the ARC,^50^ or based on the amyloid load and using of k‐means clustering and the mean change + 2SD in an Aβ negative group.^51^ Whereas these strategies tend to maximize the difference between Aβ‐accumulators and non‐accumulators, our normative approach to define Aβ‐accumulators might be helpful in identifying earlier individuals at greater risk of becoming amyloid positive.

Our study's approach to use a stable reference group for estimating longitudinal variability aligns with the recent QIBA profile^26^ and has been used in several studies.^19^, ^51^, ^52^, ^53^ However, our reference group selection criteria were stricter and included CSF amyloid and tau measurements. This could explain why in AIBL for instance, the 95^th^ percentile absolute change in an amyloid negative group (defined as CL < 20) was 6.56 CL/year (Bourgeat et al.^19^) whereas the 95^th^ percentile estimate in our study was 3.0 CL/year. Further investigations are crucial to evaluate the notion of reliable accumulation on which is base the classification of individuals as Aβ‐accumulators or non‐accumulators, considering factors such as the tracer used, the reference region, changes in scanner in between timepoints, and registration methods. Additionally, taking into account the population's diversity and the type of dataset is crucial. Indeed, reliable accumulation in curated research datasets might be lower than more heterogeneous clinical datasets. This underscores the need for robustness testing and cautious interpretation in estimating reliable accumulation in future studies.

As longitudinal PET studies using CL become increasingly widespread, establishing a standardized strategy to determine reliable accumulation and Aβ‐accumulators can help better track subthreshold amyloid accumulation and can potentially help assess potential re‐accumulation of amyloid after successful treatment.

Numerous CL thresholds have been established to correlate the scale with varying levels of amyloid pathology. Based on post‐mortem studies^30^ and CSF studies, a CL below 10 units would reliably exclude the presence of amyloid, and a CL load above 30 units would be strong evidence of the presence of amyloid.^38^ The window between 10 and 30 CL units can be regarded as a “gray zone,” indicative of an evolving pathology trending toward positivity. Indeed, in previous studies, VR‐based thresholds typically fell within this gray zone, ranging from 17 CL for expert readers^33^, ^37^ to 26 CL in several studies.^30^, ^33^, ^54^ Our findings suggest that the lower end of the gray zone (∼12–20 CL) could represent the optimal window to predict short‐term Aβ accumulation as reflected by a reliable CL increase. These results are in accordance with the work of Farrell et al. who reported an optimal threshold to predict future accumulation varying from 15 to 17.5 CL across AIBL, HABS and ADNI cohorts,^25^ as well as the reliable worsening estimate of 19 CL determined by Jack et al.^24^ (Figure 5). In the future, in a clinical setting focused on secondary prevention, a follow‐up scan could be considered after 2 years for individuals with a CL above 15 but below 30 units.

Furthermore, we established three scenarios to help inform subject selection strategies. In our precision‐recall analysis, by setting a minimum precision and recall of 0.7 and minimum specificity of 0.9, the aim was to help minimize false positives, help minimize false negatives, or increase our ability to correctly predict non‐accumulators. Increasing precision and specificity results in baseline CL thresholds higher than our reference estimate (albeit overlapping confidence intervals) and, therefore, closer to VR‐based positivity thresholds. As can be expected, increasing recall markedly decreased baseline CL threshold. Indeed, as the CL burden reflects the cumulative effect of amyloid accumulation over time, a few subjects with a low baseline amyloid burden are also Aβ‐accumulators.

Finally, in assessing the longitudinal CL trajectories over time, two primary factors emerged as influential on the ARC: APOE‐ε4 carriership and level of education. Although we found a significant impact of APOE‐ε4 carriership on the ARC, this might not be generalizable to cohorts with higher amyloid burden or mostly cognitively impaired individuals.^23^, ^55^, ^56^ Indeed, compared to non‐carriers, APOE‐ε4 carriers are more likely to accumulate Aβ pathology and tend to develop the disease earlier.^57^, ^58^ In addition, the level of education is sometimes used as a proxy for conceptualizing resistance to amyloid deposition^59^, ^60^; however, further studies with more specific markers are warranted to elucidate the potential protective factors against amyloid accumulation. Importantly, our results showed no differences in longitudinal trajectories across tracers, confirming that the CL scale is well‐suited for multi‐tracer, longitudinal PET studies. Finally, no difference was observed between cognitive groups, which probably reflects that the PNHS (like Insight46) is a preclinical cohort with only 5% of individuals having (very mild) cognitive impairment.

The current study also presents some limitations. First, VR were performed by local readers, so some disagreement is to be expected. Second, the CL is derived from the SUVr, which is a semi‐quantitative measure that could be affected by some treatment strategies (e.g., blood flow fluctuations, reference kinetics, tracer clearance). Modifications in these factors will lead to changes in SUVr, independent of any shifts in amyloid levels. Therefore, future trials should reassess the validity of SUVr for each new drug using dynamic PET to perform a full kinetic analysis. Third, the definition of reliable accumulation is linked to the methodology employed to calculate the ARC. If the ARC was determined based on LME estimates, we would expect lower values. Last, our reference subset demonstrated a bias toward FMM, incorporating only 15 FBB scans. Similarly, the Insight46 reference subset consisted of 35 individuals only. To refine our understanding of reliable accumulation, future evaluations should be conducted per tracer and encompass datasets with larger sample sizes.

The present study characterized Aβ accumulation expressed in CL units using three United States Food and Drug Administration and European Medicines Agency approved fluorinated amyloid tracers in a mainly pre‐clinical population. We first presented a normative strategy to define reliable amyloid accumulation by estimating the variability of longitudinal CL measurements (3 CL/year in the PNHS) in a sub‐population expected to remain stable over time. We then established a baseline CL of 16 [12,19] to help predict future Aβ‐accumulators. Our results support a CL window of 12–20 for inclusion of subjects into early secondary prevention studies.

## CONFLICT OF INTEREST STATEMENT

A.B., C.B., and G.F. are employees of GE HealthCare. L.C. has received research support from GE HealthCare (paid to institution). D.A. received funding by the Swiss National Science Foundation (project CRSK‐3_196354/1). OG‐R receives research support from F. Hoffmann‐La Roche Ltd. and has given lectures in symposia sponsored by Roche Diagnostics. M.B. received research funding from the Instituto de Salud Carlos III (ISCIII) Acción Estratégica en Salud, integrated in the Spanish National RCDCI Plan and financed by ISCIII‐Subdirección General de Evaluación and the Fondo Europeo de Desarrollo Regional (FEDER‐Una manera de hacer Europa) grant PI17/01474, and the European Union/EFPIA Innovative Medicines Initiative Joint MOPEAD project (grant number 115985). M.M. reports research funding from the Instituto de Salud Carlos III (ISCIII) Acción Estratégica en Salud, integrated in the Spanish National RCDCI Plan and financed by ISCIII‐Subdirección General de Evaluación and the Fondo Europeo de Desarrollo Regional (FEDER—Una manera de hacer Europa) grant PI19/00335, travel support from F. Hoffmann‐La Roche Ltd. and participation on the Spanish Scientific Advisory Board for biomarkers from Araclon‐biotech—Grífols. M.Sch. has served on scientific advisory boards for Servier Pharmaceuticals, NovoNordisk and Roche, and has received funding from Roche, Novo Nordisk and Bioarctic (paid to institution), all outside the scope of this study. RW is an employee of IXICO. J.M.S. has received research funding and PET tracer from AVID Radiopharmaceuticals (a wholly owned subsidiary of Eli Lilly) and Alliance Medical; has consulted for Roche, Eli Lilly, Biogen, AVID, Merck, and GE; and received royalties from Oxford University Press and Henry Stewart Talks. He is Chief Medical Officer for Alzheimer's Research UK, and Medical Advisor to UK Dementia Research Institute. R.G. is a full‐time employee of Life Molecular Imaging GmbH, Berlin, Germany. A.D. reports the following: Research support from Siemens Healthineers, Life Molecular Imaging, GE Healthcare, AVID Radiopharmaceuticals, Sofie, Eisai, Novartis/AAA, Ariceum Therapeutics; Speaker Honorary/Advisory Boards: Siemens Healthineers, Sanofi, GE Healthcare, Biogen, Novo Nordisk, Invicro, Novartis/AAA, Bayer Vital; Stock: Siemens Healthineers, Lantheus Holding, Structured therapeutics, ImmunoGen; Patents: Patent for 18F‐JK‐PSMA‐ 7 (PSMA PET imaging tracer, Patent No.: EP3765097A1; Date of patent: Jan. 20, 2021). J.D.G. reports research support from GE Healthcare, Roche Diagnostics, and Hoffmann‐La Roche; has given lectures in symposia sponsored by General Electrics, Philips Nederlands, Life Molecular Imaging, and Biogen; has served on scientific advisory boards or as a consultant for Prothena Biosciences and Roche Diagnostics; and is the inventor, founder, and co‐owner of BetaScreen. F.B. is a steering committee or iDMC member for Biogen, Merck, Roche, EISAI and Prothena. He is a consultant for Roche, Biogen, Merck, IXICO, Jansen, Combinostics and has research agreements with Merck, Biogen, GE Healthcare, Roche. He is co‐founder and shareholder of Queen Square Analytics LTD. The remaining authors have no conflicts of interest to declare. Author disclosures are available in the Supporting information.

## CONSENT STATEMENT

All human subjects provided informed consent.

## Supporting information

Supporting Information
